# A Case of COVID-19 Mimicking Acute Appendicitis in Multi-System Inflammatory Syndrome

**DOI:** 10.7759/cureus.15600

**Published:** 2021-06-11

**Authors:** Anna Martin, Taylor Otto, Travis Smith

**Affiliations:** 1 College of Osteopathic Medicine, Lake Erie College of Osteopathic Medicine (LECOM) - Bradenton, Bradenton, USA; 2 Clinical Curriculum Integration & Assessment, Lake Erie College of Osteopathic Medicine (LECOM) - Bradenton, Bradenton, USA

**Keywords:** multi-system inflammatory syndrome in children (mis-c), sars-cov-2, covid-19

## Abstract

Children’s naive immune systems allow for a unique course of the novel severe acute respiratory syndrome coronavirus 2 (SARS-CoV-2) virus when compared to adults. In multi-system inflammatory syndrome in children (MIS-C), a current or recent SARS-CoV-2 infection can cause fever and elevated inflammatory markers in individuals under the age of 21. Similar to Kawasaki disease, Kikuchi disease, systemic lupus erythematosus, toxic shock syndrome (TSS), and macrophage activation syndrome (MAS), there is an influx of inflammation associated with MIS-C that creates this pathologic state. Because MIS-C affects numerous organ systems, its presentation varies substantially, thus making it difficult to diagnose and treat in a timely fashion. In our case, a previously healthy four-year-old African American female initially presented to the emergency department (ED) with high fever, abdominal pain, and headache after recent SARS-Co-V-2 exposure. After initially being diagnosed with a urinary tract infection (UTI), she returned with a myriad of symptoms, including persistent fever, abdominal pain, and conjunctivitis. Her initial SARS-CoV-2 test returned positive, and she was admitted and placed on broad-spectrum antibiotics then requiring vasopressors, mechanical ventilation, and an appendectomy. Her workup revealed elevated inflammatory markers, elevated brain natriuretic peptide (BNP), anemia, thrombocytopenia, pyuria, and hypercoagulability meeting the criteria for MIS-C. In addition to antibiotics, her treatment included IV immunoglobulin and methylprednisolone until the patient was stabilized for discharge. As more is learned about SARS-CoV-2, it will become increasingly important to consider the development and implications of MIS-C. Educating providers on the wide range of MIS-C presentations can lead to more effective preventative measures and treatments.

## Introduction

Out of all severe acute respiratory syndrome coronavirus 2 (SARS-CoV-2) infections, 7.3% of cases are in children, and those numbers are expected to increase [1]. As of March 2021, a total of 3,185 cases of multi-system inflammatory syndrome in children (MIS-C) have been reported in the United States [2]. Specifically, MIS-C has been shown to affect children and lead to a more severe disease state. MIS-C is associated with fever, inflammatory gastrointestinal (GI) symptoms, conjunctivitis, and dermatological manifestations [3]. The range of progression of this disease shows a wide variety of presentations as it can rapidly approach, causing hypotension and severe respiratory distress. Becoming familiar with this condition and its association with coronavirus disease 2019 (COVID-19) will be helpful as recognizing and promptly treating MIS-C can help prevent its progression and a possible ominous outcome.

## Case presentation

A previously healthy four-year-old African American female presented to the emergency department (ED) with a one-day history of persistent fever, malaise, anorexia, and headache. On physical exam, she was found to be in no acute distress. She was tachycardic with a temperature of 39.5°C and had mild pharyngeal erythema. An initial rapid strep test returned negative and a COVID-19 nasopharyngeal swab was obtained because the patient endorsed recent exposure, but the results were delayed. Urinalysis revealed pyuria leading to the initial diagnosis of a urinary tract infection (UTI), and she was discharged on cephalexin. A chest radiograph was taken at this time that revealed bronchiolitic changes consistent with COVID-19, but no specific treatment such as inhaled corticosteroids or remdesivir was administered due to the absence of any respiratory symptoms. Two days later, the patient returned to the ED with persistent high fever (38.7°C), new-onset mild vague abdominal pain, diarrhea (without nausea or vomiting), dark urine, and bilateral conjunctivitis. Repeat urinalysis showed persistent pyuria, but the urine culture from the previous visit showed no growth, and cephalexin was discontinued. Her COVID-19 polymerase chain reaction (PCR) test from the prior visit returned positive, and a repeat chest radiograph continued to show bronchiolitic changes though she did not have any clinical symptoms such as dyspnea or cough. Complete blood count (CBC) and comprehensive metabolic panel (CMP) revealed mild anemia (hemoglobin, Hgb 10.3 g/dL, hematocrit, Hct 30.4%) and thrombocytopenia (127,000/mcL) without leukocytosis or electrolyte abnormalities. A coagulation panel revealed normal prothrombin time (PT) and INR but elevated partial thromboplastin time (PTT) (40.5 s), fibrinogen (588 mg/dL), and D-dimer (1.81 mcg/mL). Inflammatory markers, other than ferritin, were elevated with an erythrocyte sedimentation rate (ESR) 50 mm/h and C-reactive protein (CRP) 16.28 mg/dL indicating a categorically high cardiovascular risk. Troponin-I levels were within normal limits, but both brain natriuretic peptide (BNP) and procalcitonin were elevated at 245 pg/mL and 3.15 ng/mL, respectively. Collectively, these labs suggested a diagnosis of acute COVID-19 with MIS-C features, though markers are not exclusive. Notably, a repeat PCR test on this date returned negative, but the patient was positive for IgG antibodies.

Acetaminophen, ibuprofen, and fluids were administered in the ED, but the patient remained tachycardic and her blood pressure dropped from 98/70 to 87/49 mmHg. Ceftriaxone was initiated for suspected sepsis and she was admitted to the floor. However, the patient remained hypotensive prompting the administration of fluid boluses and the replacement of ceftriaxone with more broad-spectrum antibiotics cefepime and vancomycin. When the patient showed increased work of breathing and signs of respiratory failure, she was transferred to the pediatric ICU where a norepinephrine drip was maintained. A chest radiograph showed new bilateral haziness and airspace opacities, and she was placed on bilevel positive airway pressure.

Due to the novelty and complexity of the case, a multidisciplinary team was consulted and involved throughout treatment in accordance with MIS-C treatment recommendations [4]. She was given intravenous immune globulin (IVIG) and methylprednisolone, and an echocardiogram was ordered revealing mild left atrial dilation with normal biventricular size and systolic function. Infectious disease specialists recommended close monitoring of inflammatory markers, and hematology-oncology specialists recommended subcutaneous enoxaparin sodium. 

On day two of hospitalization, her abdominal pain worsened prompting an abdominal ultrasound that revealed a small amount of free fluid and a slightly edematous gallbladder. The patient also developed right hip pain, and an ultrasound revealed trace effusion. A subsequent increase in ESR (55 mm/h) and CRP (20 mg/dL) suggested an increase in overall inflammation due to her condition. Likewise, her previously normal troponin-I increased to 0.08 ng/mL, and BNP rose to 1433 pg/mL. Over the next day, these lab values remained elevated, and additional labs reflected a neutrophil-predominant increase in white blood cells from 7,200 to 11,000/mcL and increased D-dimer of 3.15 mcg/mL. Her systolic blood pressures remained in the 80s and she became progressively more tachycardic and tachypneic with breakthrough fevers.

**Figure 1:**
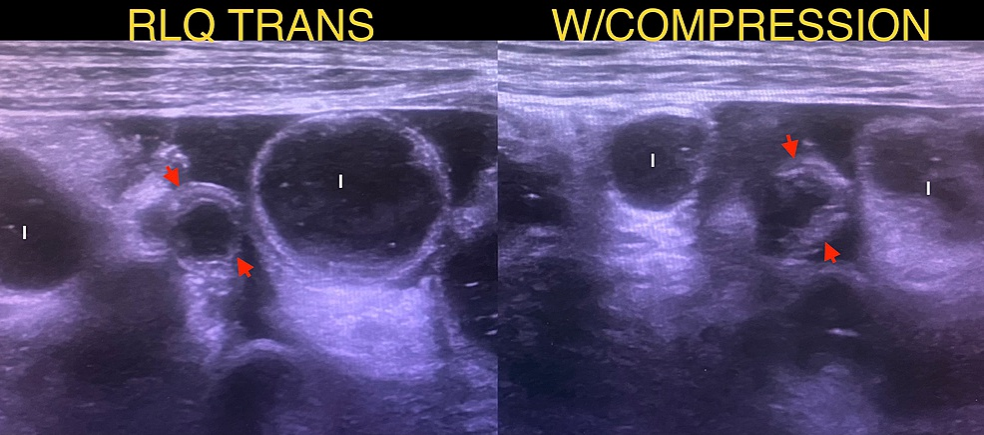
Abdominal ultrasound suggestive of acute appendicitis on hospitalization day 4. Appendix enlarged in diameter with inflamed and uneven walls (red arrows) with surrounding exudation zone between intestinal loops (I).

By day four of hospitalization, the abdominal pain had become more severe and localized to her right lower quadrant. The CBC now showed leukocytosis (white blood cell count, WBC 19,600/mcL) with 80% neutrophils and 12% band neutrophils and a critical drop in Hgb (6.7 g/dL) and Hct (19.9%). Coags were in range, and she was transfused appropriately. Chest radiograph showed left lower lobe consolidation consistent with atelectasis, and an abdominal ultrasound was repeated and showed a fluid-filled, noncompressible appendix dilated up to 7 mm, suspicious for acute appendicitis (Figure 1). The ultrasound also revealed debris in the urinary bladder, clinically correlating her pyuria with her initially diagnosed UTI. Enoxaparin sodium was held, and laparoscopic appendectomy was performed without complication. The retrocecal appendix appeared grossly normal with minimal induration or neovascularity, but the patient had significant ascites and ileus, presumably secondary to COVID-19. The intact appendix was sent to pathology in formalin and measured 5.4 cm long and 0.6 cm in diameter without a distinct rupture site. Microscopic analysis with hematoxylin and eosin stain revealed purple-pink serosa and congested vasculature, and sectioning revealed a patent lumen with dark red fluid and a wall thickness averaging 0.2 cm.

Following her appendectomy bilevel positive airway pressure (BiPAP) was continued, and a repeat chest radiograph revealed increased left lower lobe consolidation with small possible pleural effusion on the right. Antibiotics were discontinued due to negative urine and sputum culture. Post-operatively, WBCs returned to normal (10,000/mcL) and Hgb and Hct remained stable at 10.4 g/dL and 32.4%, respectively. CRP, BNP, and troponin-I remained elevated (15 mg/dL, 751 pg/mL, and 0.06 ng/mL). 

On hospitalization day 5, a repeat ultrasound of the right hip revealed decreased trace joint effusion with new synovial thickening but no drainable fluid collection. Chest radiograph continued to show persistent hazy opacities with possible bilateral pleural effusion. The patient remained hemodynamically stable without pressor support and was transferred back to the floor with the continuation of steroids and IVIG. 

The patient continued to feel better and on hospitalization day 6 troponin-I normalized (0.05 ng/mL). On hospitalization day 7, BNP peaked at 1564 pg/mL before dropping to 141 pg/mL the following day. An echocardiogram revealed normal systolic function, a trivial rim of pericardial fluid, and very mild left atrial dilation. 

Over the next two days, a neutrophil-predominant leukocytosis returned (21,500 and 15,600/mcL) and Hgb and Hct decreased to critical values (6.4 g/dL and 19.7%). D-dimer was elevated (14.05 vs. 6.93 mcg/mL the previous day), and a chest radiograph was performed revealing improved aeration of lung bases without indications of a new pathology. Due to concern of blood loss at the surgical site, an abdominal ultrasound was performed suggesting the presence of a hematoma overlying the bladder amidst a moderate amount of free fluid. At this time, enoxaparin sodium was stopped, and on hospitalization day 9 Hgb and Hct increased to 10.1 g/dL and 30.7%. The patient continued to have pain and guarding of her lower abdomen prompted concern for expansion of the suprapubic hematoma. On hospitalization day 11, ultrasound findings revealed a mild decrease in overall size, and lab values improved. 

Over hospitalization days 10-14, the patient continued to improve clinically. D-dimer, ferritin, CRP, and BNP remained elevated but stable (7.83 mcg/mL; 387 ng/mL; 1.1 mg/dL; and 270 pg/mL), and leukocytes, PT, and PTT normalized. The patient was discharged home with oral steroids for two weeks and was advised to follow up with rheumatology and cardiology. At follow-up, her only complaint was nonspecific abdominal pain. Repeat abdominal imaging was not performed, but repeat labs showed that her Hgb was improving, now up to 11.1 g/dL, along with a declining ferritin level of 217 ng/mL, with her CRP and D-dimer levels now returning to normal. Cardiology confirmed that a subsequent echocardiogram was normal, and rheumatology’s only further recommendations were to hold all vaccines for three to four months and live vaccines for 11 months since her illness.

## Discussion

Multi-system inflammatory syndrome in children (MIS-C) from COVID-19 was first described in April 2020 in the UK, followed by Italy and New York [3]. In May 2020, the Centers for Disease Control and Prevention (CDC) defined criteria for a reportable case of MIS-C as an individual under the age of 21 years with a minimum of 24-h subjective or objective fever ≥38℃ or higher; severe illness necessitating hospitalization; involvement of two or more organ systems; lab evidence of inflammation [at least one of the following: elevated CRP, ESR, procalcitonin, fibrinogen, D-dimer, ferritin, lactate dehydrogenase (LDH), interleukin-6 (IL-6), neutrophils, or low albumin]; either positive SARS-CoV-2 testing by reverse transcriptase-polymerase chain reaction (RT-PCR), antigen, or serology or COVID-19 exposure within the recent four-weeks prior to symptom onset; and no alternative diagnosis [3]. Temporal and epidemiologic evidence suggests that MIS-C is strongly associated with SARS-CoV-2, though causality has not been confirmed [3]. Most cases of MIS-C have been RT-PCR negative but antibody positive, indicating prior infection [3, 5-6]. Interestingly, our case demonstrates a rapid progression of symptoms from the time the patient originally tested positive to the time she tested negative (with antibodies) two days later, in support of the theory that MIS-C is a post-infectious inflammatory immune dysregulation [3, 5-6].

Emerging studies are beginning to reveal more specific commonalities among cases of MIS-C, many of which also apply to our case. For instance, several studies have suggested that the incidence of MIS-C is higher in black populations, and most individuals with MIS-C did not have any prior comorbidities [3, 5-9]. Importantly, gastrointestinal symptoms ranging from abdominal pain and vomiting to hepatitis and pancreatitis were prominent in the majority of reported cases [5, 7-10]. Conversely, this case differs from the majority of reported cases in that this patient was female and slightly younger than the average patient with MIS-C. Systematic reviews to date have consistently reported that most patients with MIS-C are male and that the median age ranges from 8.6 to 10 [5, 9].

Only one other case report has described appendicitis in the context of MIS-C [8]. In this case, the patient was a few years older and presented after three days of fever, with negative PCR but positive immunoglobulin G (IgG) antibodies. Ultrasound revealed a 9 mm-thick non-compressible appendix with an appendicolith at the base of the cecum. Whereas our patient had a laparoscopic appendectomy without complications, this patient had undergone open appendectomy for additional resection of inflamed ileum with yellow surrounding purulent peritoneal fluid. Both patients’ appendices were congested and inflamed, but hyperplastic lymphoid tissue and necrotizing lymphadenitis with necrotic foci were not found in our patient as they were in the other case study, as well as several other cases of MIS-C [3]. More research is needed to thoroughly explain the pathogenesis of COVID-19 with respect to the lymphatic and gastrointestinal systems, but it is currently believed that the angiotensin-converting enzyme 2 (ACE2) receptor may play a role, as it is expressed on the gastrointestinal (GI) tract mucosa and integral to virus’ entry into cells [11]. Importantly, histologic findings have proven non-specific; thus clinical correlation is imperative for diagnoses in treatment [8]. Some diseases that share several overlapping symptoms and histologies include Kawasaki disease, Kikuchi disease, systemic lupus erythematosus, toxic shock syndrome (TSS), and macrophage activation syndrome (MAS) [3-4, 8-9]. However, a syndrome of persistent fever and GI symptoms with elevated pro-BNP and CRP and neutrophilia with lymphopenia are some of the most pathognomonic signs for MIS-C [3-4, 8-9].

Overall, our patient’s course of MIS-C was not atypical compared to other described cases. She presented sooner after symptom onset (one day vs. five-day average) and was hospitalized for a total of 13 days (vs. eight-day average), including ICU stay (71% of cases) [7]. Thus her total course of illness was approximately 14-15 days, as seen in most cases. 

The goal of the multidisciplinary approach toward treatment was to dampen inflammation and cytokine storm [4]. The standard treatment options include treatment for dehydration, fever, hypercoagulability, and stress ulcer prophylaxis. The treatment with immune globulin (1-2 g/kg, once daily) and methylprednisolone (1-2 mg/kg, divided twice daily) are the current recommendations in the treatment for MIS-C [4]. Though infusion reactions, anaphylaxis, transaminitis, hemolysis, hypertension, and hyperglycemia have been noted in some in response to this treatment, our patient did not experience any of these complications [4, 8]. With continued treatment, our patient improved clinically with her laboratory studies reflected her declining inflammatory response. Her response to this treatment reflects the majority of outcomes as most recover rapidly after this combination of therapy, but those who do not can try other alternative treatments such as anakinra, a recombinant IL-1B antagonist [3, 9].

## Conclusions

Ultimately, much remains to be elucidated regarding the pathogenesis of MIS-C and its long-term sequelae. With the ongoing pandemic involving COVID-19, long-term effects and acute reactions to the virus need to be studied meticulously. As shown from this case review, acute appendicitis can be a unique symptom of MIS-C associated with COVID-19. It is important for clinicians to recognize that the viral inflammatory process can present with abdominal pain and subsequent inflammation of the appendix so that it can be diagnosed and treated early in the course of the disease to help prevent systemic decline. Even among reported cases of MIS-C, appendicitis and its treatment may differ in their course and outcomes. Furthermore, the additional focus should be placed on post-operative management of acute appendicitis in the context of MIS-C-related hemodynamic instability to better provide insight to future healthcare teams treating similar patients. Analysis of more reported cases of appendicitis in the context of MIS-C should aid in identifying both preventative and therapeutic strategies to standardize a more efficacious care plan for children affected by this illness.
